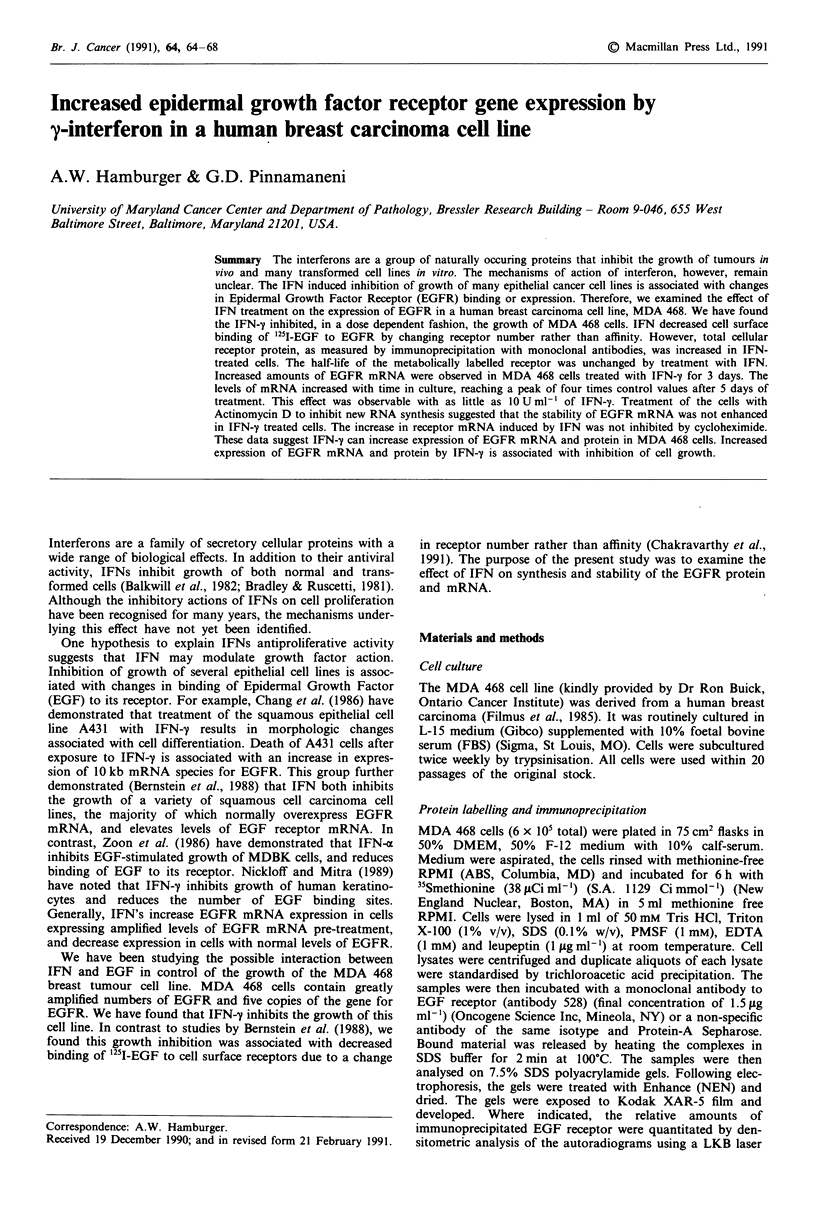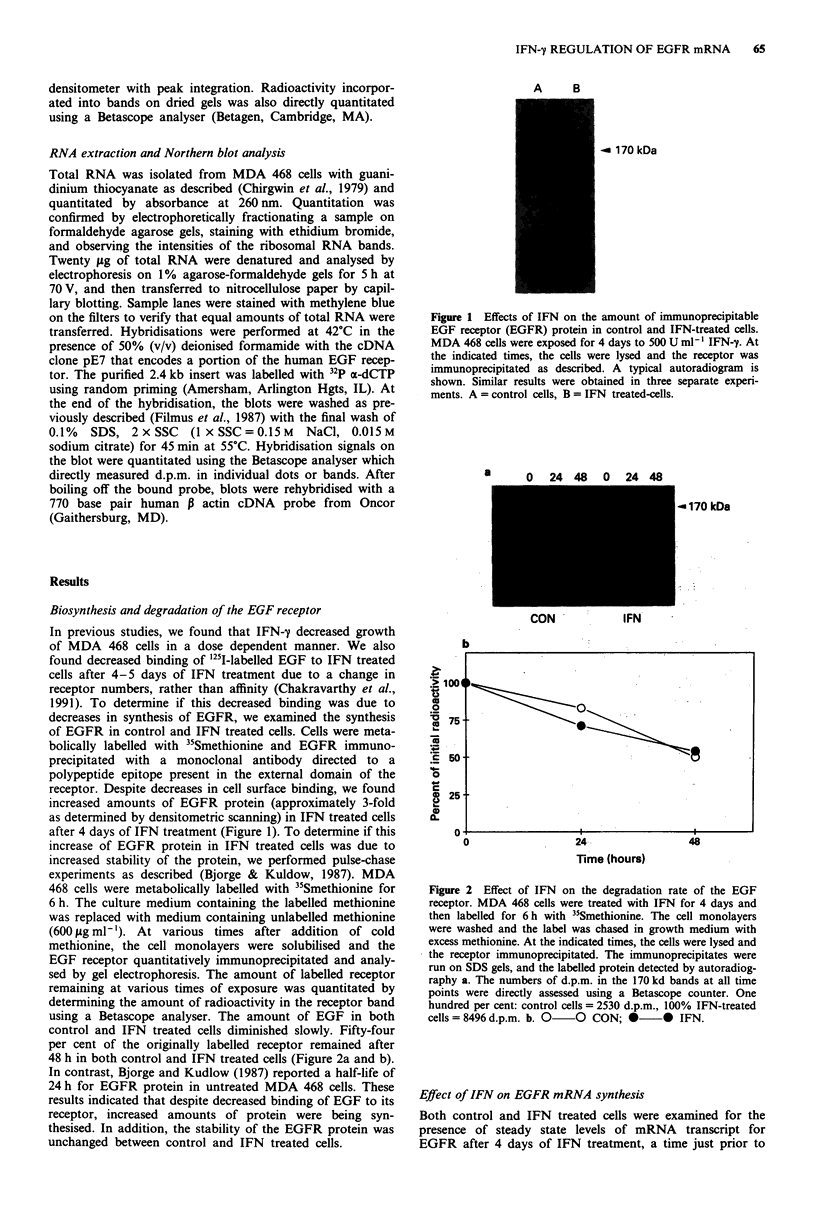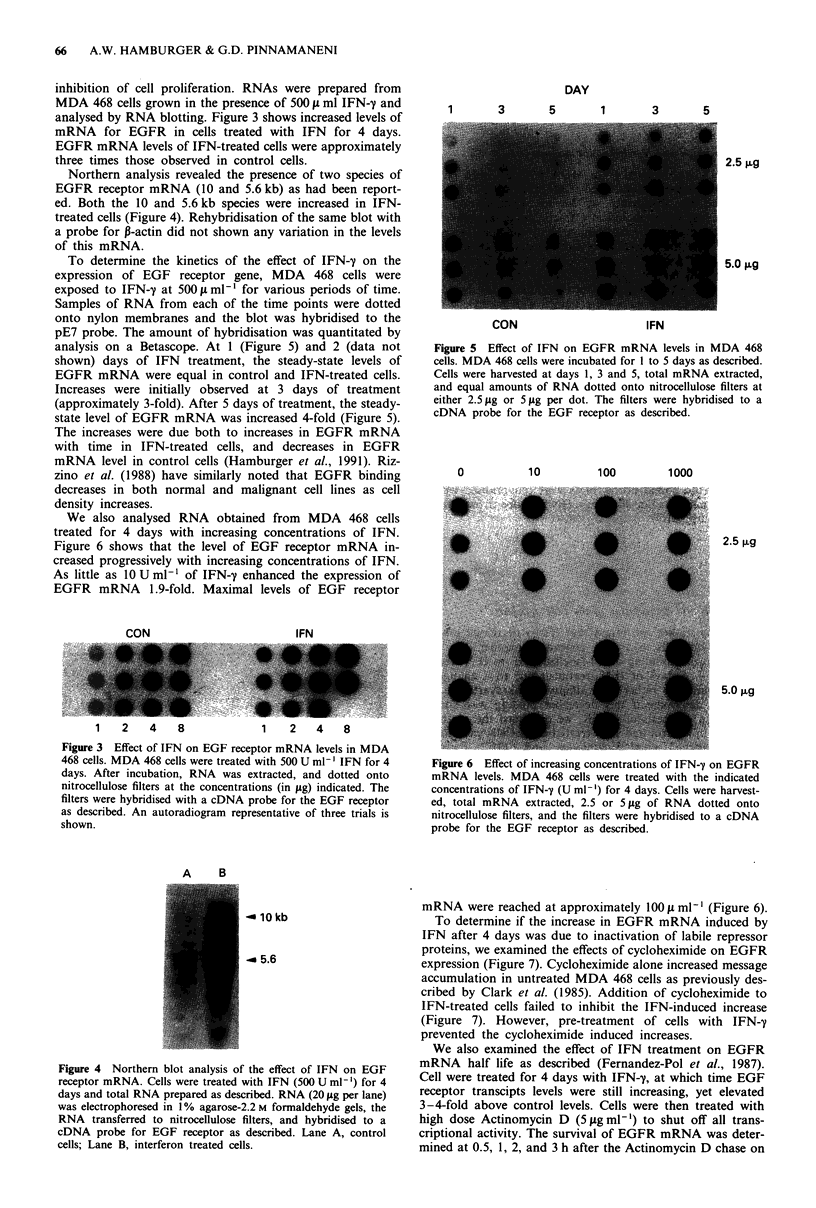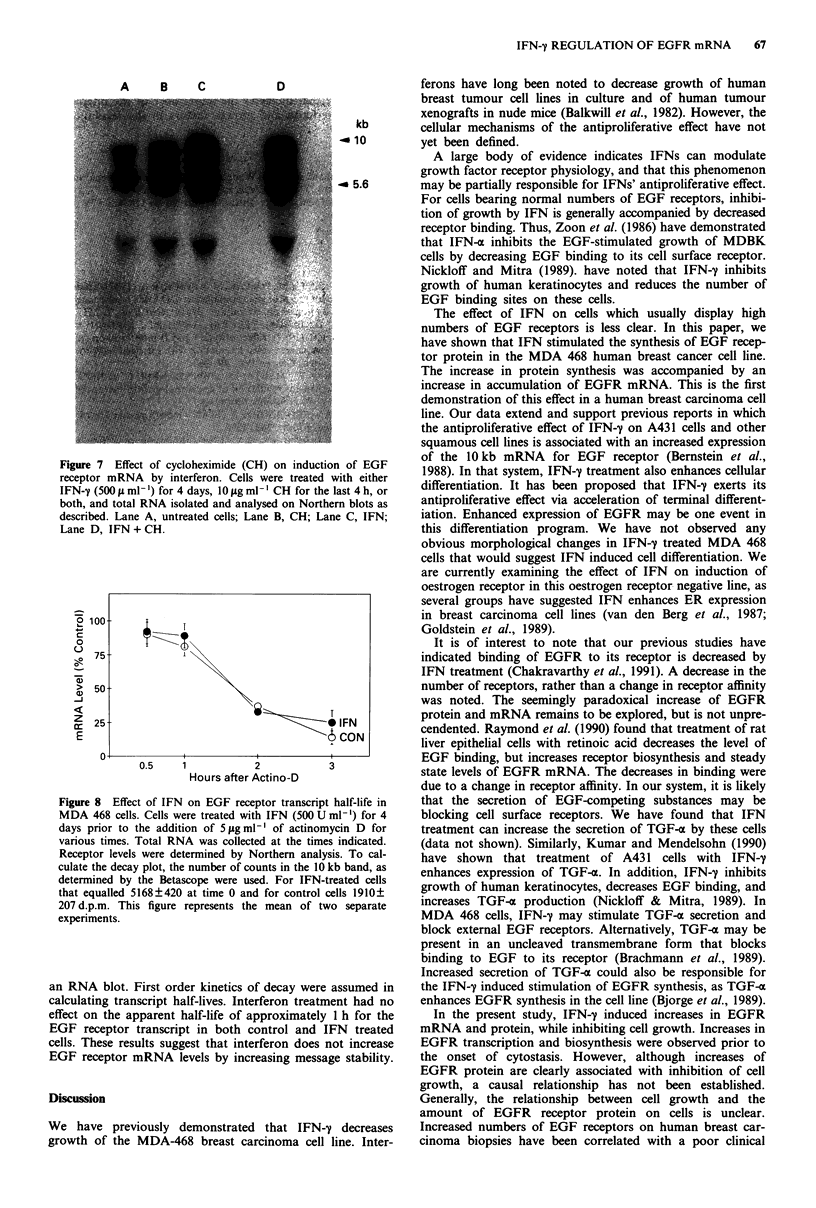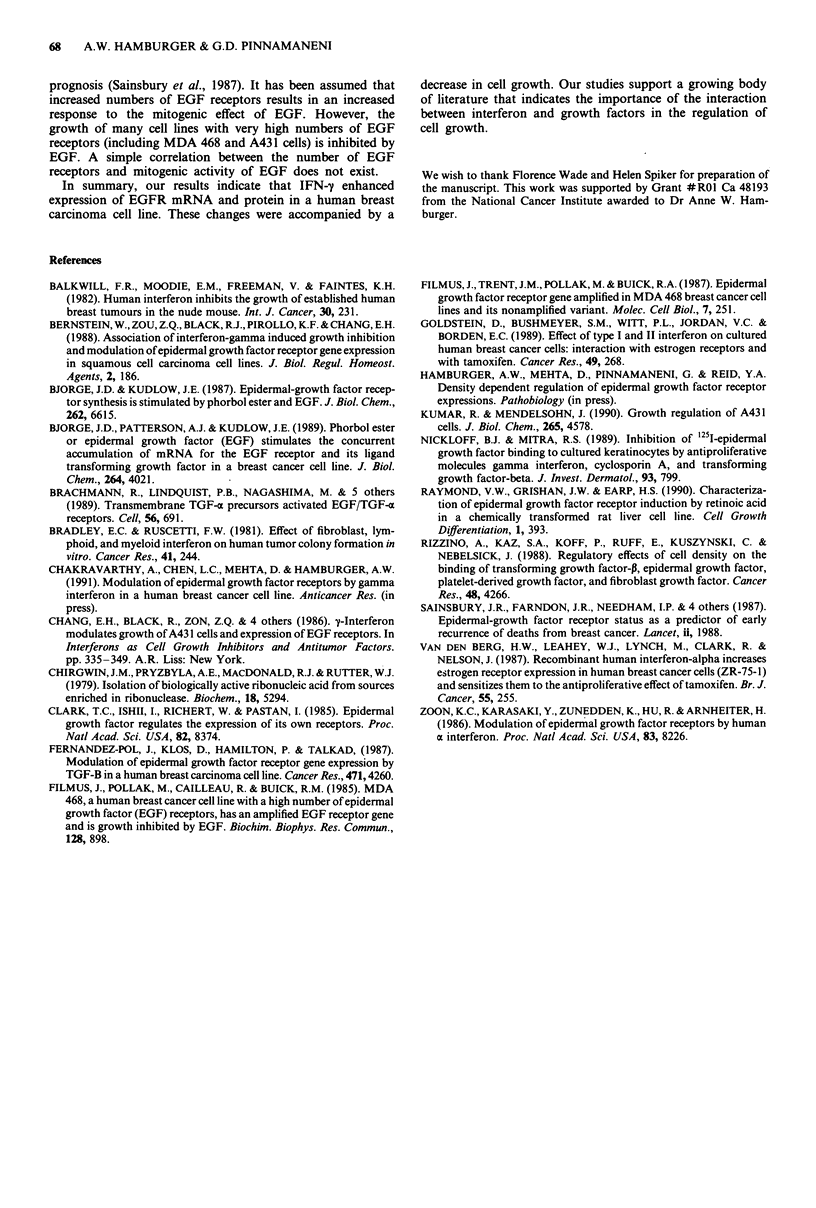# Increased epidermal growth factor receptor gene expression by gamma-interferon in a human breast carcinoma cell line.

**DOI:** 10.1038/bjc.1991.240

**Published:** 1991-07

**Authors:** A. W. Hamburger, G. D. Pinnamaneni

**Affiliations:** University of Maryland Cancer Center, Baltimore 21201.

## Abstract

**Images:**


Br. J. Cancer (1991), 64, 64–68 © Macmillan Press Ltd., 1991

### Increased epidermal growth factor receptor gene expression by γ-interferon in a human breast carcinoma cell line

A.W. Hamburger & G.D. Pinnamaneni

*University of Maryland Cancer Center and Department of Pathology, Bressler Research Building – Room 9-046, 655 West Baltimore Street, Baltimore, Maryland 21201, USA.*

**Summary** The interferons are a group of naturally occuring proteins that inhibit the growth of tumours *in vivo* and many transformed cell lines *in vitro*. The mechanisms of action of interferon, however, remain unclear. The IFN induced inhibition of growth of many epithelial cancer cell lines is associated with changes in Epidermal Growth Factor Receptor (EGFR) binding or expression. Therefore, we examined the effect of IFN treatment on the expression of EGFR in a human breast carcinoma cell line, MDA 468. We have found the IFN-γ inhibited, in a dose dependent fashion, the growth of MDA 468 cells. IFN decreased cell surface binding of $^{125}$I-EGF to EGFR by changing receptor number rather than affinity. However, total cellular receptor protein, as measured by immunoprecipitation with monoclonal antibodies, was increased in IFN-treated cells. The half-life of the metabolically labelled receptor was unchanged by treatment with IFN. Increased amounts of EGFR mRNA were observed in MDA 468 cells treated with IFN-γ for 3 days. The levels of mRNA increased with time in culture, reaching a peak of four times control values after 5 days of treatment. This effect was observable with as little as 10 U ml$^{-1}$ of IFN-γ. Treatment of the cells with Actinomycin D to inhibit new RNA synthesis suggested that the stability of EGFR mRNA was not enhanced in IFN-γ treated cells. The increase in receptor mRNA induced by IFN was not inhibited by cycloheximide. These data suggest IFN-γ can increase expression of EGFR mRNA and protein in MDA 468 cells. Increased expression of EGFR mRNA and protein by IFN-γ is associated with inhibition of cell growth.

Interferons are a family of secretory cellular proteins with a wide range of biological effects. In addition to their antiviral activity, IFNs inhibit growth of both normal and transformed cells (Balkwill *et al.*, 1982; Bradley & Ruscetti, 1981). Although the inhibitory actions of IFNs on cell proliferation have been recognised for many years, the mechanisms underlying this effect have not yet been identified.

One hypothesis to explain IFNs antiproliferative activity suggests that IFN may modulate growth factor action. Inhibition of growth of several epithelial cell lines is associated with changes in binding of Epidermal Growth Factor (EGF) to its receptor. For example, Chang *et al.* (1986) have demonstrated that treatment of the squamous epithelial cell line A431 with IFN-γ results in morphologic changes associated with cell differentiation. Death of A431 cells after exposure to IFN-γ is associated with an increase in expression of 10 kb mRNA species for EGFR. This group further demonstrated (Bernstein *et al.*, 1988) that IFN both inhibits the growth of a variety of squamous cell carcinoma cell lines, the majority of which normally overexpress EGFR mRNA, and elevates levels of EGF receptor mRNA. In contrast, Zoon *et al.* (1986) have demonstrated that IFN-α inhibits EGF-stimulated growth of MDBK cells, and reduces binding of EGF to its receptor. Nickloff and Mitra (1989) have noted that IFN-γ inhibits growth of human keratinocytes and reduces the number of EGF binding sites. Generally, IFN's increase EGFR mRNA expression in cells expressing amplified levels of EGFR mRNA pre-treatment, and decrease expression in cells with normal levels of EGFR.

We have been studying the possible interaction between IFN and EGF in control of the growth of the MDA 468 breast tumour cell line. MDA 468 cells contain greatly amplified numbers of EGFR and five copies of the gene for EGFR. We have found that IFN-γ inhibits the growth of this cell line. In contrast to studies by Bernstein *et al.* (1988), we found this growth inhibition was associated with decreased binding of $^{125}$I-EGF to cell surface receptors due to a change in receptor number rather than affinity (Chakravarthy *et al.*, 1991). The purpose of the present study was to examine the effect of IFN on synthesis and stability of the EGFR protein and mRNA.

Correspondence: A.W. Hamburger.
Received 19 December 1990; and in revised form 21 February 1991.

### Materials and methods

### *Cell culture*

The MDA 468 cell line (kindly provided by Dr Ron Buick, Ontario Cancer Institute) was derived from a human breast carcinoma (Filmus *et al.*, 1985). It was routinely cultured in L-15 medium (Gibco) supplemented with 10% foetal bovine serum (FBS) (Sigma, St Louis, MO). Cells were subcultured twice weekly by trypsinisation. All cells were used within 20 passages of the original stock.

### *Protein labelling and immunoprecipitation*

MDA 468 cells ($6 \times 10^5$ total) were plated in 75 cm$^2$ flasks in 50% DMEM, 50% F-12 medium with 10% calf-serum. Medium were aspirated, the cells rinsed with methionine-free RPMI (ABS, Columbia, MD) and incubated for 6 h with $^{35}$Smethionine (38 μCi ml$^{-1}$) (S.A. 1129 Ci mmol$^{-1}$) (New England Nuclear, Boston, MA) in 5 ml methionine free RPMI. Cells were lysed in 1 ml of 50 mM Tris HCl, Triton X-100 (1% v/v), SDS (0.1% w/v), PMSF (1 mM), EDTA (1 mM) and leupeptin (1 μg ml$^{-1}$) at room temperature. Cell lysates were centrifuged and duplicate aliquots of each lysate were standardised by trichloroacetic acid precipitation. The samples were then incubated with a monoclonal antibody to EGF receptor (antibody 528) (final concentration of 1.5 μg ml$^{-1}$) (Oncogene Science Inc, Mineola, NY) or a non-specific antibody of the same isotype and Protein-A Sepharose. Bound material was released by heating the complexes in SDS buffer for 2 min at 100°C. The samples were then analysed on 7.5% SDS polyacrylamide gels. Following electrophoresis, the gels were treated with Enhance (NEN) and dried. The gels were exposed to Kodak XAR-5 film and developed. Where indicated, the relative amounts of immunoprecipitated EGF receptor were quantitated by densitometric analysis of the autoradiograms using a LKB laser densitometer with peak integration. Radioactivity incorporated into bands on dried gels was also directly quantitated using a Betascope analyser (Betagen, Cambridge, MA).

*RNA extraction and Northern blot analysis*

Total RNA was isolated from MDA 468 cells with guanidinium thiocyanate as described (Chirgwin *et al.*, 1979) and quantitated by absorbance at 260 nm. Quantitation was confirmed by electrophoretically fractionating a sample on formaldehyde agarose gels, staining with ethidium bromide, and observing the intensities of the ribosomal RNA bands. Twenty μg of total RNA were denatured and analysed by electrophoresis on 1% agarose-formaldehyde gels for 5 h at 70 V, and then transferred to nitrocellulose paper by capillary blotting. Sample lanes were stained with methylene blue on the filters to verify that equal amounts of total RNA were transferred. Hybridisations were performed at 42°C in the presence of 50% (v/v) deionised formamide with the cDNA clone pE7 that encodes a portion of the human EGF receptor. The purified 2.4 kb insert was labelled with $^{32}P$ α-dCTP using random priming (Amersham, Arlington Hgts, IL). At the end of the hybridisation, the blots were washed as previously described (Filmus *et al.*, 1987) with the final wash of 0.1% SDS, 2 × SSC (1 × SSC = 0.15 M NaCl, 0.015 M sodium citrate) for 45 min at 55°C. Hybridisation signals on the blot were quantitated using the Betascope analyser which directly measured d.p.m. in individual dots or bands. After boiling off the bound probe, blots were rehybridised with a 770 base pair human β actin cDNA probe from Oncor (Gaithersburg, MD).

### Results

*Biosynthesis and degradation of the EGF receptor*

In previous studies, we found that IFN-γ decreased growth of MDA 468 cells in a dose dependent manner. We also found decreased binding of $^{125}I$-labelled EGF to IFN treated cells after 4–5 days of IFN treatment due to a change in receptor numbers, rather than affinity (Chakravarthy *et al.*, 1991). To determine if this decreased binding was due to decreases in synthesis of EGFR, we examined the synthesis of EGFR in control and IFN treated cells. Cells were metabolically labelled with $^{35}S$methionine and EGFR immunoprecipitated with a monoclonal antibody directed to a polypeptide epitope present in the external domain of the receptor. Despite decreases in cell surface binding, we found increased amounts of EGFR protein (approximately 3-fold as determined by densitometric scanning) in IFN treated cells after 4 days of IFN treatment (Figure 1). To determine if this increase of EGFR protein in IFN treated cells was due to increased stability of the protein, we performed pulse-chase experiments as described (Bjorge & Kuldow, 1987). MDA 468 cells were metabolically labelled with $^{35}S$methionine for 6 h. The culture medium containing the labelled methionine was replaced with medium containing unlabelled methionine (600 μg $ml^{-1}$). At various times after addition of cold methionine, the cell monolayers were solubilised and the EGF receptor quantitatively immunoprecipitated and analysed by gel electrophoresis. The amount of labelled receptor remaining at various times of exposure was quantitated by determining the amount of radioactivity in the receptor band using a Betascope analyser. The amount of EGF in both control and IFN treated cells diminished slowly. Fifty-four per cent of the originally labelled receptor remained after 48 h in both control and IFN treated cells (Figure 2a and b). In contrast, Bjorge and Kudlow (1987) reported a half-life of 24 h for EGFR protein in untreated MDA 468 cells. These results indicated that despite decreased binding of EGF to its receptor, increased amounts of protein were being synthesised. In addition, the stability of the EGFR protein was unchanged between control and IFN treated cells.

**Figure 1** Effects of IFN on the amount of immunoprecipitable EGF receptor (EGFR) protein in control and IFN-treated cells. MDA 468 cells were exposed for 4 days to 500 U $ml^{-1}$ IFN-γ. At the indicated times, the cells were lysed and the receptor was immunoprecipitated as described. A typical autoradiogram is shown. Similar results were obtained in three separate experiments. A = control cells, B = IFN treated-cells.

**Figure 2** Effect of IFN on the degradation rate of the EGF receptor. MDA 468 cells were treated with IFN for 4 days and then labelled for 6 h with $^{35}S$methionine. The cell monolayers were washed and the label was chased in growth medium with excess methionine. At the indicated times, the cells were lysed and the receptor immunoprecipitated. The immunoprecipitates were run on SDS gels, and the labelled protein detected by autoradiography **a**. The numbers of d.p.m. in the 170 kd bands at all time points were directly assessed using a Betascope counter. One hundred per cent: control cells = 2530 d.p.m., 100% IFN-treated cells = 8496 d.p.m. **b**. O——O CON; ●——● IFN.

*Effect of IFN on EGFR mRNA synthesis*

Both control and IFN treated cells were examined for the presence of steady state levels of mRNA transcript for EGFR after 4 days of IFN treatment, a time just prior to inhibition of cell proliferation. RNAs were prepared from MDA 468 cells grown in the presence of 500 $\mu$ ml IFN-$\gamma$ and analysed by RNA blotting. Figure 3 shows increased levels of mRNA for EGFR in cells treated with IFN for 4 days. EGFR mRNA levels of IFN-treated cells were approximately three times those observed in control cells.

Northern analysis revealed the presence of two species of EGFR receptor mRNA (10 and 5.6 kb) as had been reported. Both the 10 and 5.6 kb species were increased in IFN-treated cells (Figure 4). Rehybridisation of the same blot with a probe for $\beta$-actin did not shown any variation in the levels of this mRNA.

To determine the kinetics of the effect of IFN-$\gamma$ on the expression of EGF receptor gene, MDA 468 cells were exposed to IFN-$\gamma$ at 500 $\mu$ ml$^{-1}$ for various periods of time. Samples of RNA from each of the time points were dotted onto nylon membranes and the blot was hybridised to the pE7 probe. The amount of hybridisation was quantitated by analysis on a Betascope. At 1 (Figure 5) and 2 (data not shown) days of IFN treatment, the steady-state levels of EGFR mRNA were equal in control and IFN-treated cells. Increases were initially observed at 3 days of treatment (approximately 3-fold). After 5 days of treatment, the steady-state level of EGFR mRNA was increased 4-fold (Figure 5). The increases were due both to increases in EGFR mRNA with time in IFN-treated cells, and decreases in EGFR mRNA level in control cells (Hamburger *et al.*, 1991). Rizzino *et al.* (1988) have similarly noted that EGFR binding decreases in both normal and malignant cell lines as cell density increases.

We also analysed RNA obtained from MDA 468 cells treated for 4 days with increasing concentrations of IFN. Figure 6 shows that the level of EGF receptor mRNA increased progressively with increasing concentrations of IFN. As little as 10 U ml$^{-1}$ of IFN-$\gamma$ enhanced the expression of EGFR mRNA 1.9-fold. Maximal levels of EGF receptor

**Figure 5** Effect of IFN on EGFR mRNA levels in MDA 468 cells. MDA 468 cells were incubated for 1 to 5 days as described. Cells were harvested at days 1, 3 and 5, total mRNA extracted, and equal amounts of RNA dotted onto nitrocellulose filters at either 2.5 $\mu$g or 5 $\mu$g per dot. The filters were hybridised to a cDNA probe for the EGF receptor as described.

**Figure 6** Effect of increasing concentrations of IFN-$\gamma$ on EGFR mRNA levels. MDA 468 cells were treated with the indicated concentrations of IFN-$\gamma$ (U ml$^{-1}$) for 4 days. Cells were harvested, total mRNA extracted, 2.5 or 5 $\mu$g of RNA dotted onto nitrocellulose filters, and the filters were hybridised to a cDNA probe for the EGF receptor as described.

**Figure 3** Effect of IFN on EGF receptor mRNA levels in MDA 468 cells. MDA 468 cells were treated with 500 U ml$^{-1}$ IFN for 4 days. After incubation, RNA was extracted, and dotted onto nitrocellulose filters at the concentrations (in $\mu$g) indicated. The filters were hybridised with a cDNA probe for the EGF receptor as described. An autoradiogram representative of three trials is shown.

**Figure 4** Northern blot analysis of the effect of IFN on EGF receptor mRNA. Cells were treated with IFN (500 U ml$^{-1}$) for 4 days and total RNA prepared as described. RNA (20 $\mu$g per lane) was electrophoresed in 1% agarose-2.2 M formaldehyde gels, the RNA transferred to nitrocellulose filters, and hybridised to a cDNA probe for EGF receptor as described. Lane A, control cells; Lane B, interferon treated cells.

mRNA were reached at approximately 100 $\mu$ ml$^{-1}$ (Figure 6).

To determine if the increase in EGFR mRNA induced by IFN after 4 days was due to inactivation of labile repressor proteins, we examined the effects of cycloheximide on EGFR expression (Figure 7). Cycloheximide alone increased message accumulation in untreated MDA 468 cells as previously described by Clark *et al.* (1985). Addition of cycloheximide to IFN-treated cells failed to inhibit the IFN-induced increase (Figure 7). However, pre-treatment of cells with IFN-$\gamma$ prevented the cycloheximide induced increases.

We also examined the effect of IFN treatment on EGFR mRNA half life as described (Fernandez-Pol *et al.*, 1987). Cell were treated for 4 days with IFN-$\gamma$, at which time EGF receptor transcipts levels were still increasing, yet elevated 3–4-fold above control levels. Cells were then treated with high dose Actinomycin D (5 $\mu$g ml$^{-1}$) to shut off all transcriptional activity. The survival of EGFR mRNA was determined at 0.5, 1, 2, and 3 h after the Actinomycin D chase on

**Figure 7** Effect of cycloheximide (CH) on induction of EGF receptor mRNA by interferon. Cells were treated with either IFN-γ (500 $\mu$ ml$^{-1}$) for 4 days, 10 $\mu$g ml$^{-1}$ CH for the last 4 h, or both, and total RNA isolated and analysed on Northern blots as described. Lane A, untreated cells; Lane B, CH; Lane C, IFN; Lane D, IFN + CH.

**Figure 8** Effect of IFN on EGF receptor transcript half-life in MDA 468 cells. Cells were treated with IFN (500 U ml$^{-1}$) for 4 days prior to the addition of 5 $\mu$g ml$^{-1}$ of actinomycin D for various times. Total RNA was collected at the times indicated. Receptor levels were determined by Northern analysis. To calculate the decay plot, the number of counts in the 10 kb band, as determined by the Betascope were used. For IFN-treated cells that equalled 5168 $\pm$ 420 at time 0 and for control cells 1910 $\pm$ 207 d.p.m. This figure represents the mean of two separate experiments.

an RNA blot. First order kinetics of decay were assumed in calculating transcript half-lives. Interferon treatment had no effect on the apparent half-life of approximately 1 h for the EGF receptor transcript in both control and IFN treated cells. These results suggest that interferon does not increase EGF receptor mRNA levels by increasing message stability.

### Discussion

We have previously demonstrated that IFN-γ decreases growth of the MDA-468 breast carcinoma cell line. Interferons have long been noted to decrease growth of human breast tumour cell lines in culture and of human tumour xenografts in nude mice (Balkwill *et al.*, 1982). However, the cellular mechanisms of the antiproliferative effect have not yet been defined.

A large body of evidence indicates IFNs can modulate growth factor receptor physiology, and that this phenomenon may be partially responsible for IFNs' antiproliferative effect. For cells bearing normal numbers of EGF receptors, inhibition of growth by IFN is generally accompanied by decreased receptor binding. Thus, Zoon *et al.* (1986) have demonstrated that IFN-α inhibits the EGF-stimulated growth of MDBK cells by decreasing EGF binding to its cell surface receptor. Nickloff and Mitra (1989). have noted that IFN-γ inhibits growth of human keratinocytes and reduces the number of EGF binding sites on these cells.

The effect of IFN on cells which usually display high numbers of EGF receptors is less clear. In this paper, we have shown that IFN stimulated the synthesis of EGF receptor protein in the MDA 468 human breast cancer cell line. The increase in protein synthesis was accompanied by an increase in accumulation of EGFR mRNA. This is the first demonstration of this effect in a human breast carcinoma cell line. Our data extend and support previous reports in which the antiproliferative effect of IFN-γ on A431 cells and other squamous cell lines is associated with an increased expression of the 10 kb mRNA for EGF receptor (Bernstein *et al.*, 1988). In that system, IFN-γ treatment also enhances cellular differentiation. It has been proposed that IFN-γ exerts its antiproliferative effect via acceleration of terminal differentiation. Enhanced expression of EGFR may be one event in this differentiation program. We have not observed any obvious morphological changes in IFN-γ treated MDA 468 cells that would suggest IFN induced cell differentiation. We are currently examining the effect of IFN on induction of oestrogen receptor in this oestrogen receptor negative line, as several groups have suggested IFN enhances ER expression in breast carcinoma cell lines (van den Berg *et al.*, 1987; Goldstein *et al.*, 1989).

It is of interest to note that our previous studies have indicated binding of EGFR to its receptor is decreased by IFN treatment (Chakravarthy *et al.*, 1991). A decrease in the number of receptors, rather than a change in receptor affinity was noted. The seemingly paradoxical increase of EGFR protein and mRNA remains to be explored, but is not unprecendented. Raymond *et al.* (1990) found that treatment of rat liver epithelial cells with retinoic acid decreases the level of EGF binding, but increases receptor biosynthesis and steady state levels of EGFR mRNA. The decreases in binding were due to a change in receptor affinity. In our system, it is likely that the secretion of EGF-competing substances may be blocking cell surface receptors. We have found that IFN treatment can increase the secretion of TGF-α by these cells (data not shown). Similarly, Kumar and Mendelsohn (1990) have shown that treatment of A431 cells with IFN-γ enhances expression of TGF-α. In addition, IFN-γ inhibits growth of human keratinocytes, decreases EGF binding, and increases TGF-α production (Nickloff & Mitra, 1989). In MDA 468 cells, IFN-γ may stimulate TGF-α secretion and block external EGF receptors. Alternatively, TGF-α may be present in an uncleaved transmembrane form that blocks binding to EGF to its receptor (Brachmann *et al.*, 1989). Increased secretion of TGF-α could also be responsible for the IFN-γ induced stimulation of EGFR synthesis, as TGF-α enhances EGFR synthesis in the cell line (Bjorge *et al.*, 1989).

In the present study, IFN-γ induced increases in EGFR mRNA and protein, while inhibiting cell growth. Increases in EGFR transcription and biosynthesis were observed prior to the onset of cytostasis. However, although increases of EGFR protein are clearly associated with inhibition of cell growth, a causal relationship has not been established. Generally, the relationship between cell growth and the amount of EGFR receptor protein on cells is unclear. Increased numbers of EGF receptors on human breast carcinoma biopsies have been correlated with a poor clinical prognosis (Sainsbury *et al.*, 1987). It has been assumed that increased numbers of EGF receptors results in an increased response to the mitogenic effect of EGF. However, the growth of many cell lines with very high numbers of EGF receptors (including MDA 468 and A431 cells) is inhibited by EGF. A simple correlation between the number of EGF receptors and mitogenic activity of EGF does not exist.

In summary, our results indicate that IFN-γ enhanced expression of EGFR mRNA and protein in a human breast carcinoma cell line. These changes were accompanied by a decrease in cell growth. Our studies support a growing body of literature that indicates the importance of the interaction between interferon and growth factors in the regulation of cell growth.

We wish to thank Florence Wade and Helen Spiker for preparation of the manuscript. This work was supported by Grant #R01 Ca 48193 from the National Cancer Institute awarded to Dr Anne W. Hamburger.

### References

BALKWILL, F.R., MOODIE, E.M., FREEMAN, V. & FAINTES, K.H. (1982). Human interferon inhibits the growth of established human breast tumours in the nude mouse. *Int. J. Cancer*, **30**, 231.

BERNSTEIN, W., ZOU, Z.Q., BLACK, R.J., PIROLLO, K.F. & CHANG, E.H. (1988). Association of interferon-gamma induced growth inhibition and modulation of epidermal growth factor receptor gene expression in squamous cell carcinoma cell lines. *J. Biol. Regul. Homeost. Agents*, **2**, 186.

BJORGE, J.D. & KUDLOW, J.E. (1987). Epidermal-growth factor receptor synthesis is stimulated by phorbol ester and EGF. *J. Biol. Chem.*, **262**, 6615.

BJORGE, J.D., PATTERSON, A.J. & KUDLOW, J.E. (1989). Phorbol ester or epidermal growth factor (EGF) stimulates the concurrent accumulation of mRNA for the EGF receptor and its ligand transforming growth factor in a breast cancer cell line. *J. Biol. Chem.*, **264**, 4021.

BRACHMANN, R., LINDQUIST, P.B., NAGASHIMA, M. & 5 others (1989). Transmembrane TGF-α precursors activated EGF/TGF-α receptors. *Cell*, **56**, 691.

BRADLEY, E.C. & RUSCETTI, F.W. (1981). Effect of fibroblast, lymphoid, and myeloid interferon on human tumor colony formation *in vitro*. *Cancer Res.*, **41**, 244.

CHAKRAVARTHY, A., CHEN, L.C., MEHTA, D. & HAMBURGER, A.W. (1991). Modulation of epidermal growth factor receptors by gamma interferon in a human breast cancer cell line. *Anticancer Res.* (in press).

CHANG, E.H., BLACK, R., ZON, Z.Q. & 4 others (1986). γ-Interferon modulates growth of A431 cells and expression of EGF receptors. In *Interferons as Cell Growth Inhibitors and Antitumor Factors*. pp. 335–349. A.R. Liss: New York.

CHIRGWIN, J.M., PRYZBYLA, A.E., MACDONALD, R.J. & RUTTER, W.J. (1979). Isolation of biologically active ribonucleic acid from sources enriched in ribonuclease. *Biochem.*, **18**, 5294.

CLARK, T.C., ISHII, I., RICHERT, W. & PASTAN, I. (1985). Epidermal growth factor regulates the expression of its own receptors. *Proc. Natl Acad. Sci. USA*, **82**, 8374.

FERNANDEZ-POL, J., KLOS, D., HAMILTON, P. & TALKAD, (1987). Modulation of epidermal growth factor receptor gene expression by TGF-B in a human breast carcinoma cell line. *Cancer Res.*, **471**, 4260.

FILMUS, J., POLLAK, M., CAILLEAU, R. & BUICK, R.M. (1985). MDA 468, a human breast cancer cell line with a high number of epidermal growth factor (EGF) receptors, has an amplified EGF receptor gene and is growth inhibited by EGF. *Biochim. Biophys. Res. Commun.*, **128**, 898.

FILMUS, J., TRENT, J.M., POLLAK, M. & BUICK, R.A. (1987). Epidermal growth factor receptor gene amplified in MDA 468 breast cancer cell lines and its nonamplified variant. *Molec. Cell Biol.*, **7**, 251.

GOLDSTEIN, D., BUSHMEYER, S.M., WITT, P.L., JORDAN, V.C. & BORDEN, E.C. (1989). Effect of type I and II interferon on cultured human breast cancer cells: interaction with estrogen receptors and with tamoxifen. *Cancer Res.*, **49**, 268.

HAMBURGER, A.W., MEHTA, D., PINNAMANENI, G. & REID, Y.A. Density dependent regulation of epidermal growth factor receptor expressions. *Pathobiology* (in press).

KUMAR, R. & MENDELSOHN, J. (1990). Growth regulation of A431 cells. *J. Biol. Chem.*, **265**, 4578.

NICKLOFF, B.J. & MITRA, R.S. (1989). Inhibition of $^{125}$I-epidermal growth factor binding to cultured keratinocytes by antiproliferative molecules gamma interferon, cyclosporin A, and transforming growth factor-beta. *J. Invest. Dermatol.*, **93**, 799.

RAYMOND, V.W., GRISHAN, J.W. & EARP, H.S. (1990). Characterization of epidermal growth factor receptor induction by retinoic acid in a chemically transformed rat liver cell line. *Cell Growth Differentiation*, **1**, 393.

RIZZINO, A., KAZ, S.A., KOFF, P., RUFF, E., KUSZYNSKI, C. & NEBELSICK, J. (1988). Regulatory effects of cell density on the binding of transforming growth factor-β, epidermal growth factor, platelet-derived growth factor, and fibroblast growth factor. *Cancer Res.*, **48**, 4266.

SAINSBURY, J.R., FARNDON, J.R., NEEDHAM, I.P. & 4 others (1987). Epidermal-growth factor receptor status as a predictor of early recurrence of deaths from breast cancer. *Lancet*, **ii**, 1988.

VAN DEN BERG, H.W., LEAHEY, W.J., LYNCH, M., CLARK, R. & NELSON, J. (1987). Recombinant human interferon-alpha increases estrogen receptor expression in human breast cancer cells (ZR-75-1) and sensitizes them to the antiproliferative effect of tamoxifen. *Br. J. Cancer*, **55**, 255.

ZOON, K.C., KARASAKI, Y., ZUNEDDEN, K., HU, R. & ARNHEITER, H. (1986). Modulation of epidermal growth factor receptors by human α interferon. *Proc. Natl Acad. Sci. USA*, **83**, 8226.